# Experiences of Persons With Executive Dysfunction in Disability Care Using a Social Robot to Execute Daily Tasks and Increase the Feeling of Independence: Multiple-Case Study

**DOI:** 10.2196/41313

**Published:** 2022-11-03

**Authors:** Kirstin van Dam, Marieke Gielissen, Rachelle Reijnders, Agnes van der Poel, Brigitte Boon

**Affiliations:** 1 Academy Het Dorp Arnhem Netherlands; 2 Siza Arnhem Netherlands; 3 Tranzo Tilburg University Tilburg Netherlands

**Keywords:** executive dysfunction, disability care, social robots, assistive technology, independence, daily tasks, executive function, rehabilitation, disability support, daily care, implementation

## Abstract

**Background:**

Executive functions are essential for independently navigating nearly all of our daily activities. Executive dysfunction often occurs as a result of a neurodevelopmental disorder. Persons with executive dysfunction experience challenges regarding independent execution of daily tasks. Social robots might support persons with executive dysfunction to execute daily tasks and promote their feeling of independence.

**Objective:**

This study aimed to study the impact of interacting with social robot Tessa on goal attainment in the execution of daily tasks and perceived independence of persons with executive dysfunction.

**Methods:**

In this multiple-case study, 18 participant–caregiver couples were followed up while using Tessa in the home environment for 3 months. Goal attainment on independently performing a self-determined goal was measured by the Goal Attainment Scale, and participant–caregiver couples were interviewed about their experience with their interaction with Tessa and how they perceived Tessa’s impact on their independence.

**Results:**

In total, 11 (61%) participants reached their goal after 6 weeks and maintained their goal after 3 months. During the study period, 2 participant–caregiver couples withdrew because of mismatch with Tessa. Participants set goals in the following domains: execution of household tasks; intake of food, water, or medication; being ready in time for an appointment; going to bed or getting out of bed on time; personal care; and exercise. Participants perceived that Tessa increased the feeling of independence by generating more structure, stimulation, and self-direction. Participant–caregiver couples reported that the auditive information provided by Tessa was more effective in coping with executive dysfunction compared to their initial approaches using visual information, and the use of Tessa had a positive impact on their relationship.

**Conclusions:**

This study paid ample time and attention to the implementation of a social robot in daily care practice. The encouraging findings support the use of social robot Tessa for the execution of daily tasks and increasing independence of persons with executive dysfunction in disability care.

## Introduction

### Executive Dysfunction

Executive functions are the controlling mechanisms of the brain and enable us to plan, focus attention, remember instructions, and initiate and manage multiple tasks [1]. Executive function is defined as “the overarching regulation of goal-directed, future-oriented, higher-order cognitive processes” [2]. Executive functions are needed for goal-oriented behavior and responding to novel situations or solving problems [3]. They are essential for independently navigating nearly all of our daily activities. Executive dysfunction often occurs as a result of a neurodevelopmental disorder [4-6]. Persons with executive dysfunction experience problems with organizing and planning, such as being on time for appointments, executing household chores, and remembering and transferring information [7]. Executive dysfunction may also have a significant emotional impact and can lead to stress and feelings of anxiety, frustration, and embarrassment [8]. Moreover, executive abilities are linked with functions essential for independently executing daily tasks [9,10]. As such, executive dysfunction may impact the feeling of independence in daily life [3].

### Technology to Increase Independence

Technology is increasingly used in disability care [11]. It can improve planning and memory among persons with executive dysfunction and might support the execution of daily tasks [12-14]. For example, Desideri et al [13] found that mobile devices and apps help to self-monitor attention-related behaviors while performing a task. Other studies showed that technology can be an important asset for optimizing independence for persons with acquired brain injury or intellectual disability [15,16]. Remote support services and smart home systems were found to promote independent living and enable persons to lead self-determined lives. A focus group of persons with intellectual disability, their relatives, and professional caregivers expressed that eHealth applications increased independence and provided more efficient support for daily functioning of persons with intellectual disability [17].

### Social Robots

A social robot is a specific kind of technology that has been investigated in older persons but can also be of interest for persons with executive dysfunction in disability care. Among older individuals with or without dementia, the advantages of social robots are reported in relation to self-reliance, security, and emotional well-being [18]. In disability care, social robots have been a topic of interest but are mainly implemented and researched among children with autism spectrum disorder to train social behavior [19]. Recently, there has been an increasing focus on the use of social robots for adults with intellectual disabilities [20,21]. These studies report preliminary positive effects, mainly on engagement, which Shukla et al [21] defined as “the process by which individuals involved in an interaction start, maintain and end their perceived connection to one another.”

Tessa is a social robot that has been developed in the Netherlands for and in co-creation with persons with executive dysfunction. Tessa is a low-complexity, easy-to-use robot with agenda functionalities. This study in everyday care practice examines the effect of Tessa on execution of daily tasks and perceived independence, focused on persons with self-reported executive dysfunction in disability care. The following research questions will be answered: (1) Do participants attain their goals on execution of daily tasks by interacting with Tessa? (2) How did the participants experience the interaction with Tessa and perceive her impact on their independence?

## Methods

### Design and Participants

In this multiple-case study, participant–caregiver couples were recruited via purposive sampling from a Dutch disability care organization. Persons with self-reported executive dysfunction and their professional caregivers were recruited through (1) a web-based advertisement on the intranet for employees of the care organization, (2) innovation experts within the care organization, and (3) word of mouth among colleagues. Persons expressing a care need regarding executive dysfunction were eligible to take part in the study when they (1) received care (either inpatient or ambulatory) from the care organization, (2) had legal capacity and were aged 18 years or older, (3) were able to follow verbal instructions, and (4) understood the Dutch language. Participants used Tessa in their home environment for 3 months. The participant–caregiver couple was assessed at 3 time points: at the start before using Tessa (T0), halfway after using Tessa for 1.5 months (T1), and at the end of the study period (T2).

### Ethical Considerations

This study was exempted from ethical approval by the Medical Ethical Review Committee of Utrecht (19/549). Participants were provided verbal and written information about the study, and informed consent was obtained prior to start of the study.

### Materials and Procedure

**Figure 1:**
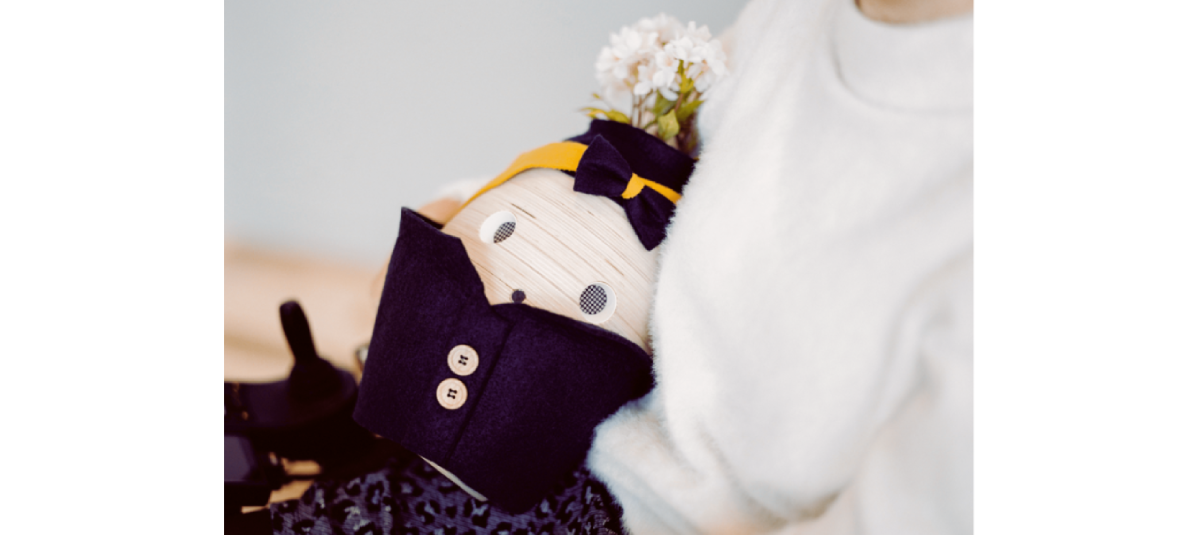
Social robot Tessa provides vocal reminders and tips at a preset time to activate and support persons with executive dysfunction in their daily activities and tasks.

Tessa is a social robot with a design that resembles a flowerpot and has a height of 30 cm, blinking light-emitting diode–lit eyes, and a female voice (see Figure 1). It is designed to take in a cozy presence in the daily living spaces of its users. Tessa runs on electricity and functions via Wi-Fi connection. She provides vocal reminders of activities, tasks, or tips at a preset time to activate and support persons with executive dysfunction. Such notifications are custom programmed in a personal web app account, for which no broad digital skills are necessary.

Social robot Tessa is distinctive from other technologies with similar purposes (eg, digital boards and apps on smartphones or tablets) because reminders are provided in the form of auditive instead of visual information. Participants of this study were interested in testing Tessa because Tessa fit their needs of receiving auditive information, which was confirmed by their caregivers.

In this study, participants used Tessa in their home environment for a period of 3 months. The researchers (KvD or RR) met with the participants at the aforementioned 3 time points during this period.

In this first visit, the following activities were carried out: (1) the researcher installed Tessa and trained the participant–caregiver couple to use it; (2) the participant–caregiver couple decided on a goal that was to be monitored during the study period, using the Goal Attainment Scale (GAS; see below); together with the researcher, they drew up the notifications belonging to this goal and programmed Tessa’s web app; (3) the participant–caregiver couples were interviewed about their expectations of using Tessa; and (4) the researcher instructed the participant–caregiver couples that in the following 3 months, they were free to change or add notifications (those related to the monitored goal or to additional goals) and that they could contact a help desk provided by the researchers in case of questions or requests for help in between contact moments.

Further, the researchers monitored a dashboard to determine whether the Tessas were active on the internet. In case a Tessa was offline for a period longer than 24 hours, the researchers contacted the corresponding participant–caregiver couple.

### Outcome Measures

#### Characteristics of Participants and Caregivers

Demographic characteristics of both participants (age, sex, and living situation) and caregivers (sex and occupation) and type of disability of the participants were assessed at T0.

#### Goal Attainment

The effect of using Tessa on independently performing a daily task was measured using the GAS [22]. At T0, each participant–caregiver couple chose 1 goal to be monitored during the research period. The researcher formulated this goal into attainment levels (decline; baseline; progress, but less than the goal; goal—see examples of the levels of attainment in Table 1) in agreement with the participant–caregiver couple. At T1 and T2, self-reported attainment of the goal was scored in accordance with the GAS (see scoring of goal attainment in Table 1). To gain insight on additional goals for which Tessa was used, we examined scripts containing the messages that the participant–caregiver couple placed in Tessa’s web app during the study period. Additional goals were not monitored.

**Table 1:** Goal attainment levels in the Goal Attainment scale, scoring, and example attainment levels of a goal (drinking 4 glasses of water in a day).

Score	Attainment^a^	Definition
–1	Decline	There is a decline compared to baseline: “Participant drinks less than two glasses of water (0.8 litres) in a day”
0	Baseline	There is no change compared to baseline: “Participant drinks two glasses of water (0.8 litres) in a day”
+1	Progress, but less than goal	There is progress, but the goal has not been attained: “Participant drinks more than two glasses, but less than four glasses (0.8-1.2 litres) of water in a day”
+2	Goal	The goal has been attained: “Participant drinks four glasses of water (1.2 litres) in a day”

^a^Two levels of the original Goal Attainment Scale were excluded (ie, “more than goal,” and “much more than goal”), as they were not applicable to the majority of the goals set and monitored in this study.

#### Participants’ Experiences and Perceived Impact on Independence

Participants were interviewed face to face at 3 time points: at T0 to focus on their expectations and at T1 and T2 to focus on their experiences of using Tessa. The main questions at T0 were the following: (1) “Why do you want to use Tessa?” (2) “How do you think Tessa will help you? What do you think will change compared to your current situation? And how would you feel about that?” The main questions asked at T1 and T2 were the following: (1) “Does Tessa help you in your daily life? And how and why is that?” (2) “Do you experience any changes since you started using Tessa? And how does that make you feel?” (3) “What do you like about Tessa and what do you not like?” (4) “How do you feel about Tessa reminding you of things compared to other people reminding you about them?” Participants were supported by their caregiver in verbally indicating their answers when needed. Caregivers shared their own insights after the participants had shared theirs. Interviews had a duration from 20 to 60 minutes each.

### Data Analysis

Data were pseudonymized and safely stored on a secure server. Descriptive statistics (frequencies and means) were used to describe the sample and GAS scores. Participants’ goals were grouped and assigned to domains. Semistructured interviews were audio recorded and transcribed verbatim with the use of pseudonyms. Data were managed with the use of Atlas.ti (version 8.4.20; ATLAS.ti Scientific Software Development GmbH) and coded separately by 2 researchers (KvD and RR). They conducted thematic analysis as an iterative process of familiarization with the data, generating initial codes, searching for and reviewing themes, and defining and naming themes [23]. Codes and themes were discussed between the 2 researchers and a third researcher (MG) until consensus was reached.

## Results

### Overview

**Figure 2:**
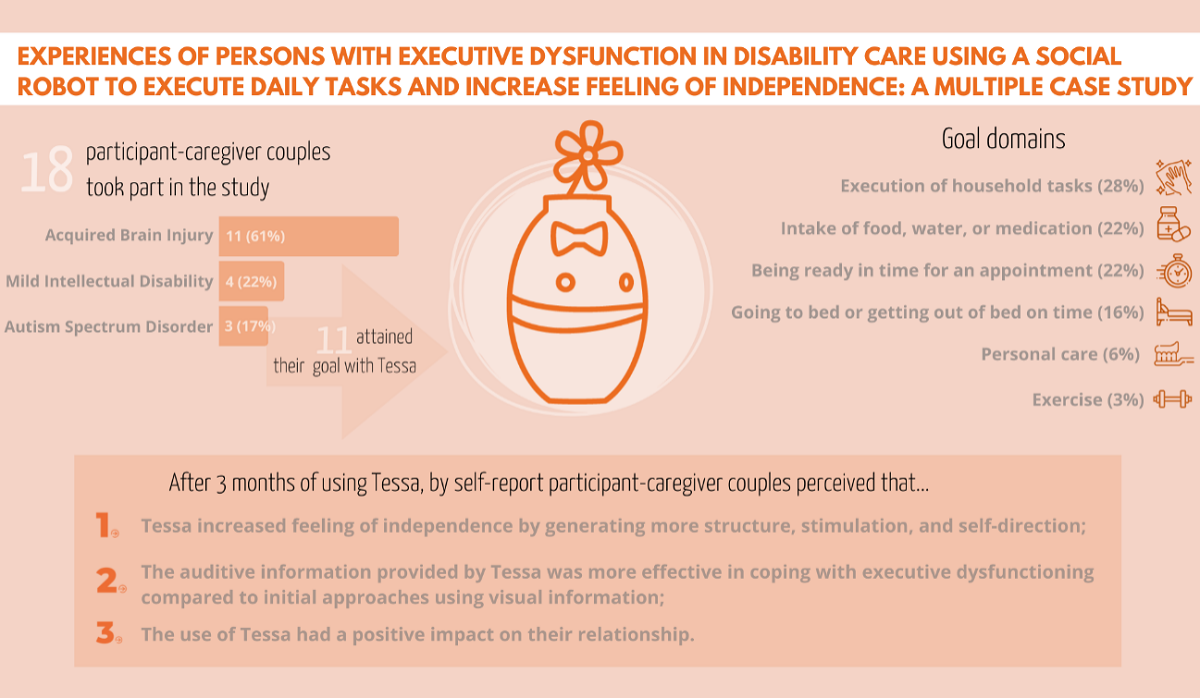
Overview of findings.

All results are visually summarized in Figure 2.

### Participants

In total, 18 participant–caregiver couples participated in the study (see Table 2). In 3 cases, a caregiver dropped out of the study owing to long-term absence or sickness. In these situations, another caregiver was asked to participate in the study. In total, 2 of 18 participant–caregiver couples withdrew early on during the study period because the participant experienced irritation or stress as a reaction to Tessa instead of feeling motivated by her. Complete data were obtained for the remaining 16 participant–caregiver couples.

**Table 2:** Characteristics of participants and caregivers.

Characteristics				Values
**Participants (n=18)**				
	Age (years), mean (range)		41 (18-63)	
	**Sex, n (%)**			
		Male	10 (56)	
		Female	8 (44)	
	**Disability, n (%)**			
		Acquired brain injury	11 (61)	
		Mild intellectual disability	4 (22)	
		Autism spectrum disorder	3 (17)	
	**Type of care, n (%)**			
		Inpatient care	8 (44)	
		Ambulatory care	10 (56)	
**Caregivers (n=17)^a^**				
	**Sex, n (%)**			
		Male	4 (24)	
		Female	13 (76)	
	**Occupation, n (%)**			
		Inpatient caregiver	8 (47)	
		Ambulatory caregiver	7 (41)	
		Cognitive therapist	2 (12)	

^a^One caregiver participated with 2 participants.

### Use and Acceptance of Tessa

The Tessa account of 3 participants was offline for a short period. Two participants did not use Tessa during the Christmas holidays—one for 1 week and the other for 2 weeks. One other participant did not use Tessa for 1 week owing to personal reasons. The Tessa accounts of all other participants were continuously in use throughout the study period.

Acceptance of Tessa is crucial for using Tessa appropriately and following her reminders. During the interviews, participants mentioned a feeling of connectedness to Tessa. Several participants stated that they thought Tessa was funny or made them happy. Some said that they viewed Tessa as a buddy or that they felt less lonely because of her presence in their home. However, at times, some participants also experienced irritation because of Tessa. When they were overstimulated or stressed already owing to other factors, these participants sometimes perceived Tessa’s notifications as unpleasant.

The funny thing is that if she asks, ‘Are you getting ready for bed?’, I react, ‘Yes, I am’, despite knowing that it’s programmed and not spontaneous. She’s got something.

Participant with an acquired brain injury

At those moments I’m really angry and I think; please, take away that doll! At one moment I had enough of it in such a way that I pulled Tessa’s plug: I am done with this.

Participant with a mild intellectual disability

### Goal Domains and Attainment

Each participant–caregiver couple set one primary goal that was monitored with the GAS. These 18 goals were grouped into 6 domains (see Table 3). Participant–caregiver couples were free to program notifications on additional goals in Tessa as well. On average, participants included 4 (range 1-8) additional goals. For these 34 additional goals (Table 4), one new domain emerged: leisure activities, such as taking time to sing, dance, or take a walk outside. The other additional goals were congruent with the previously established domains. All goals were equally distributed over domains, regardless of disability or type of care. Table 3 shows that at T1, 61% (11/18) of participants reached their primary goal. At T2, 72% (13/18) of participants were able to execute daily tasks and activities as they aspired. Out of the 5 participants who did not attain their goal, 2 withdrew owing to negative responses to Tessa, and 3 of them missed Tessa’s messages owing to them not being at home or in a different part of the house during these moments.

At T2, participants were asked whether they would like to continue using Tessa. Two participants decided to discontinue using Tessa; one of them reached their primary goal, while the other did not. Both of them had a relatively large house, which caused them to not always hear notifications, thus missing Tessa’s messages. A total of 14 participants chose to keep using Tessa; 2 of them did not yet reach their primary goal; nonetheless, they experienced support of Tessa.

**Table 3:** Domains: primary goal, disability, type of care, and Goal Attainment Scale (GAS) scores of participants at T1^a^ and T2^b^ (n=18).

Example primary goals grouped in domains		Disability	Care	GAS score at T1	GAS score at T2
**Execution of household tasks (n=5, 28%)**					
	Hand in cellphone to caregiver on time 3 times a week	Autism spectrum disorder	Inpatient	1	2
	Vacuum-clean sleeping room once a week	Autism spectrum disorder	Ambulatory	2	2
	Do the dishes every evening	Acquired brain injury	Inpatient	2	2
	Make a grocery list (in an app) once a week^d^	Acquired brain injury	Ambulatory	—^c^	—
	Bring laundry downstairs twice a week^d^	Acquired brain injury	Ambulatory	—	—
**Intake of food, water, or medication (n=4, 22%)**					
	Drink 1 glass of water in the morning	Mild intellectual disability	Inpatient	1	2
	Drink 1.2 L of water every day	Acquired brain injury	Ambulatory	2	2
	Take medicine on time 4 times a day	Mild intellectual disability	Inpatient	2	2
	Take medicine on time 3-6 times a week	Acquired brain injury	Ambulatory	2	2
**Being ready in time for an appointment (n=4, 22%)**					
	Leave on time for volunteering work once a week	Acquired brain injury	Ambulatory	2	2
	Be on time for taxi to day-care 2 days a week	Mild intellectual disability	Inpatient	2	2
	Leave on time for day-care 3 days a week	Acquired brain injury	Inpatient	1	0
	Leave on time for day-care 2 days a week	Mild intellectual disability	Ambulatory	0	0
**Going to bed or getting out of bed on time (n=3, 16%)**					
	Get out of bed within half an hour every day	Autism spectrum disorder	Inpatient	2	2
	Do night routine and go to bed on time every night	Acquired brain injury	Ambulatory	2	2
	Go to sleep before 11:15 PM every night	Acquired brain injury	Ambulatory	2	2
**Personal care (n=1, 6%)**					
	Shave in the morning every day	Acquired brain injury	Inpatient	2	2
**Exercise (n=1, 6%)**					
	Do back exercises in the home gymnasium once a week	Acquired brain injury	Ambulatory	0	0

^a^T1: 6 weeks.

^b^T2: 3 months.

^c^Not available.

^d^These participants withdrew, their goals were not monitored.

**Table 4:** Domains: additional goals of participants (n=18).

Domains	Participants, n (%)
Execution of household tasks	6 (33)
Intake of food, water, or medication	7 (39)
Being ready in time for an appointment	7 (39)
Going to bed or getting out of bed on time	5 (28)
Personal care	4 (22)
Exercise	1 (6)
Leisure activities^a^	4 (28)

^a^New domain—this domain was not reported for the primary goals.

### Perceived Impact of Tessa on Independence

Qualitative data analysis revealed that participants perceived that Tessa impacted their independence through 3 elements: structure, stimulation, and self-direction. No differences in perceived effects were found for disability and type of care.

#### Structure

Participants valued the repetitiveness of Tessa and the fixed moments of her messages, which, for some participants, even served as training to build new habits. Some participants appreciated the stability of Tessa’s fixed spot in the house. Tessa also helped participants to have a sense of time, which is something they struggled with beforehand. Participants explained that structure in their daily lives helps them to “get into a rhythm,” which gives them clarity and rest.

Very often I’m too late or much too early, because I can't estimate the time properly. Now when I have an appointment, Tessa says half an hour in advance, 'Remember, you have an appointment later. Get ready.“ This is like a helping hand.

Participant with an acquired brain injury

Sometimes we come ten minutes late or, you know, something always happens. And Tessa is always on time.

Caregiver of participant with autism spectrum disorder

#### Stimulation

Participants perceived that Tessa stimulated them to be active, which caused them to be and feel less dependent on stimulation from others to perform their daily tasks and activities. The stimuli of Tessa’s messages were an active reminder to do something. Both participants and caregivers emphasized that Tessa’s verbal instructions were far more stimulating than visual reminders (such as app notifications or sticky notes). They described Tessa’s reminders as a “wake-up call” or “hint.”

The activities programmed in Tessa are now much easier for me to execute. Now I pack my bag on time, not fifteen minutes late.

Participant with autism spectrum disorder

It’s very nice to see that verbal information makes a difference, because we have been working towards structure for years. We tried different things: an app, calendar, whiteboard… They didn't work, as he forgot to check them. Which made him miss the appointment. He pushes the notification away on his mobile, and it's done. Tessa can repeat a message and thus gives a reminder that he can't push away.

Caregiver of participant with an acquired brain injury

#### Self-direction

Participants perceived a sense of self-direction, as Tessa enables them to have more control over their lives and to make their own decisions. At T0, many participants expressed the aspiration that Tessa would help them be less reliant on reminders of others, which at T1 and T2 had actually happened. Both participants and caregivers confirmed that less unplanned contact was needed between them because of the reminders of Tessa. Participants explained how reminders of Tessa felt different than reminders of others.

What I really like about Tessa is that I put messages in it myself. Then, when Tessa says something, I know I want it myself. It feels more my own, it doesn't come from others. Instead of others telling me to do something.

Participant with autism spectrum disorder

Because Tessa helps me remember things, such as brushing my teeth, I am less irritated. It makes me feel good that caregivers no longer say it.

Participant with a mild intellectual disability

#### Caregivers

As the quotes imply, caregivers were positive about the use and effects of Tessa on participants’ independence. Moreover, caregivers explained that they experienced positive effects of Tessa on their relation with the participant. With Tessa reminding the participant, caregivers experienced less of a burden and felt less friction between them and the participants. Furthermore, instead of spending the majority of time on practical issues, they were now able to spend more time on discussing more profound topics relevant to the participant. As an example of such topics, caregivers mentioned emotional or social issues such as the participant’s stress regulation or social network.

We have more time for other things, because we’re not constantly busy with reminding. We can instead focus a bit more on support and counselling, not only the very practical daily tasks.

Caregiver of participant with autism spectrum disorder

It also relieves us a bit. Sometimes other situations have priority. When we need to be with someone who is having an epileptic seizure, for example, we can't be here to remind the participant to put on his jacket. In that moment Tessa is still reminding him, so we know it will be fine.

Caregiver of participant with a mild intellectual disorder

## Discussion

### Principal Findings

This study provides encouraging findings supporting the use of social robot Tessa for the execution of daily tasks and increasing independence of persons with executive dysfunction in disability care. In total, 18 participants stated goals in the following domains: execution of household tasks; intake of food, water, or medication; being ready in time for an appointment; going to bed or getting out of bed on time; personal care; and exercise. For additional purposes, participants used Tessa for the same domains and to remind them to perform leisure activities. A total of 11 (61%) participants reached their goal after 6 weeks (T1) and maintained their goal after 3 months (T2). Tessa increased participants’ independence by generating more structure, stimulation, and self-direction. Considering our results between the type of disability (autism spectrum disorder, acquired brain injury, and mild intellectual disability) and type of care (inpatient versus ambulant care), no differences were found in the type of goals that were stated, attainment of the primary goals, and perceived impact on independence by participants. All caregivers were positive about the use of Tessa in the care for persons with executive dysfunction. The caregivers and participants reported that the auditive information provided by Tessa was more effective in coping with executive dysfunction than their initial approaches using visual information; in addition, the use of Tessa had a positive impact on their relationship.

The findings of this study show that in the majority of cases, Tessa was suitable to reach personal goals and increase independence in persons with executive dysfunction in disability care. However, in some cases Tessa was not helpful owing to mismatch. Tessa’s messages were not always heard by participants who lived in a big house or had irregular schedules, and 2 participants stopped using Tessa as they experienced irritation or stress as a reaction to Tessa’s notifications. As preferences and needs differ from person to person, a person-oriented approach in deciding whether or not and how to deploy Tessa is essential. At the same time, the use of technologies such as Tessa may support the person-centeredness of care [24], as it initiates conversation about the goals and challenges of persons with executive dysfunction and how they want to work toward reaching or overcoming these. In using Tessa, the participant and caregiver started by setting personal goals and placing corresponding messages in Tessa, and they evaluated these together over time.

### Comparison With Prior Work

To our knowledge, this is the first study that reports on the effect of a social robot for the execution of daily tasks and independence of persons with executive dysfunction in disability care. The existing literature mainly describes the deployment of social robots in therapy and care for purposes related to support for teaching social behavior to children with autism spectrum disorder or to companionship or assistance to older individuals in the home environment [25]. Similar results were found in studies examining a different technology with the same objectives [26-29]. Several studies observed that smartphone-based systems supported persons with intellectual disability to successfully start and carry out daily activities [26-28], and O’Neill et al [29] found that interactive prompting technology reduced the support needed for the morning routine of persons with executive dysfunction due to acquired brain injury.

### Limitations

This study paid ample time and attention to the implementation of Tessa, as a good implementation process is not only important for successful dissemination of interventions but also has a major influence on the effectiveness of the intervention. Research shows that interventions that are well implemented are 2-12 times more effective [30]. Tessa was studied in daily care practice where caregivers already have a heavy workload owing to staff shortages. Hence, we attempted to minimize the burden on participant–caregiver couples. First, a resulting limitation of this study is the fact that we only used self-reported measurements. To gain more insight in the characteristics of the end-user of Tessa, future studies should include a multiple-format assessment using neuropsychological tests and self-report measures to assess participants’ level of functioning and execution of daily activities. Second, the use of purposive sampling and therefore the heterogeneity of the sample can be seen as a limitation. The sample represents the situation in the participating organization but might not be generalizable to all populations in disability care. Future studies should preferably include large homogenous populations to determine the effect and suitability of social robots for specific target groups in disability care.

### Conclusions

Technology is and will be increasingly used in disability care [31,32]. We studied the utility of social robot Tessa in disability care practice and found it to be a helpful technology for different target groups that experience challenges owing to executive dysfunction. These promising results are an example of how technology can support the independence of persons in disability care.

## Abbreviations

GAS: Goal Attainment Scale

## Data Availability

The data that support the findings of this study are available on request from the corresponding author.

## References

[1] Evans J (2003). Rehabilitation of executive deficits. Neuropsychological Rehabilitation: Theory and Practice.

[2] Demetriou EA, DeMayo MM, Guastella AJ (2019). Executive function in autism spectrum disorder: history, theoretical models, empirical findings, and potential as an endophenotype. Front Psychiatry.

[3] Chung CSY, Pollock A, Campbell T, Durward BR, Hagen S (2013). Cognitive rehabilitation for executive dysfunction in adults with stroke or other adult non-progressive acquired brain damage. Cochrane Database Syst Rev.

[4] Rabinovici GD, Stephens ML, Possin KL (2015). Executive dysfunction. Continuum (Minneap Minn).

[5] Panerai S, Tasca D, Ferri R, Genitori D'Arrigo V, Elia M (2014). Executive functions and adaptive behaviour in autism spectrum disorders with and without intellectual disability. Psychiatry J.

[6] Stuss DT (2011). Traumatic brain injury: relation to executive dysfunction and the frontal lobes. Curr Opin Neurol.

[7] ter Stal M, Patel S, de Groot V, Gielissen M, van der Poel A, Boon B (2021). Verbeteren van dagstructuur bij mensen met niet-aangeboren hersenletsel (NAH) door inzet van technologie.

[8] Larsson Lund M, Lövgren-Engström AL, Lexell J (2011). Using everyday technology to compensate for difficulties in task performance in daily life: experiences in persons with acquired brain injury and their significant others. Disabil Rehabil Assist Technol.

[9] Nguyen CM, Copeland CT, Lowe DA, Heyanka DJ, Linck JF (2020). Contribution of executive functioning to instrumental activities of daily living in older adults. Appl Neuropsychol Adult.

[10] Cahn-Weiner DA, Boyle PA, Malloy PF (2002). Tests of executive function predict instrumental activities of daily living in community-dwelling older individuals. Appl Neuropsychol.

[11] Zander V, Gustafsson C, Landerdahl Stridsberg S, Borg J (2021). Implementation of welfare technology: a systematic review of barriers and facilitators. Disabil Rehabil Assist Technol.

[12] Torra Moreno M, Canals Sans J, Colomina Fosch MT (2021). Behavioral and cognitive interventions with digital devices in subjects with intellectual disability: a systematic review. Front Psychiatry.

[13] Desideri L, Lancioni G, Malavasi M, Gherardini A, Cesario L (2020). Step-instruction technology to help people with intellectual and other disabilities perform multistep tasks: a literature review. J Dev Phys Disabil.

[14] Gillespie A, Best C, O'Neill B (2012). Cognitive function and assistive technology for cognition: a systematic review. J Int Neuropsychol Soc.

[15] Jamwal R, Jarman HK, Roseingrave E, Douglas J, Winkler D (2022). Smart home and communication technology for people with disability: a scoping review. Disabil Rehabil Assist Technol.

[16] Tassé MJ, Wagner JB, Kim M (2020). Using technology and remote support services to promote independent living of adults with intellectual disability and related developmental disabilities. J Appl Res Intellect Disabil.

[17] Frielink N, Oudshoorn CEM, Embregts PJCM (2020). eHealth in support for daily functioning of people with intellectual disability: Views of service users, relatives, and professionals on both its advantages and disadvantages and its facilitating and impeding factors. J Intellect Dev Disabil.

[18] Góngora Alonso S, Hamrioui S, de la Torre Díez I, Motta Cruz E, López-Coronado M, Franco M (2019). Social robots for people with aging and dementia: a systematic review of literature. Telemed J E Health.

[19] Kumazaki H, Muramatsu T, Yoshikawa Y, Matsumoto Y, Ishiguro H, Kikuchi M, Sumiyoshi T, Mimura M (2020). Optimal robot for intervention for individuals with autism spectrum disorders. Psychiatry Clin Neurosci.

[20] Mitchell A, Sitbon L, Balasuriya S, Koplick S, Beaumont C (2021). Social robots in learning experiences of adults with intellectual disability: an exploratory study.

[21] Shukla J, Cristiano J, Oliver J, Puig D (2019). Robot assisted interventions for individuals with intellectual disabilities: impact on users and caregivers. Int J of Soc Robotics.

[22] Barbieri T, Fraternali P, Bianchi A, Tacchella C (2010). Autonomamente: using goal attainment scales to evaluate the impact of a multimodal domotic system to support autonomous life of people with cognitive impairment.

[23] Braun V, Clarke V (2006). Using thematic analysis in psychology. Qual Res Psychol.

[24] Chu CH, Ronquillo C, Khan S, Hung L, Boscart V (2021). Technology recommendations to support person-centered care in long-term care homes during the COVID-19 pandemic and beyond. J Aging Soc Policy.

[25] Cifuentes CA, Pinto MJ, Céspedes N, Múnera M (2020). Social robots in therapy and care. Curr Robot Rep.

[26] Lancioni GE, Singh NN, O'Reilly MF, Sigafoos J, Alberti G, Chiariello V, Buono S (2020). Extended smartphone-aided program to sustain daily activities, communication and leisure in individuals with intellectual and sensory-motor disabilities. Res Dev Disabil.

[27] Lancioni GE, Singh NN, O'Reilly MF, Sigafoos J, Alberti G, Del Gaudio V, Abbatantuono C, Taurisano P, Desideri L (2022). People with intellectual and sensory disabilities can independently start and perform functional daily activities with the support of simple technology. PLoS One.

[28] Resta E, Brunone L, D'Amico F, Desideri L (2021). Evaluating a low-cost technology to enable people with intellectual disability or psychiatric disorders to initiate and perform functional daily activities. Int J Environ Res Public Health.

[29] OʼNeill B, Best C, OʼNeill L, Ramos SDS, Gillespie A (2018). Efficacy of a micro-prompting technology in reducing support needed by people with severe acquired brain injury in activities of daily living: a randomized control trial. J Head Trauma Rehabil.

[30] Durlak JA, DuPre EP (2008). Implementation matters: a review of research on the influence of implementation on program outcomes and the factors affecting implementation. Am J Community Psychol.

[31] Lancioni GE, Olivetti Belardinelli M, Singh NN, O'Reilly MF, Sigafoos J, Alberti G (2019). Recent technology-aided programs to support adaptive responses, functional activities, and leisure and communication in people with significant disabilities. Front Neurol.

[32] (2021). Prevalence of coverage of assistive technology in the European Region: a scoping review. World Health Organization. Regional Office for Europe.

